# Identification of an Effective Early Signaling Signature during Neo-Vasculogenesis *In Vivo* by *Ex Vivo* Proteomic Profiling

**DOI:** 10.1371/journal.pone.0066909

**Published:** 2013-06-24

**Authors:** Rokhsareh Rohban, Andreas Reinisch, Nathalie Etchart, Katharina Schallmoser, Nicole A. Hofmann, Krisztina Szoke, Jan E. Brinchmann, Ehsan Bonyadi Rad, Eva Rohde, Dirk Strunk

**Affiliations:** 1 Stem Cell Research Unit, Medical University of Graz, Graz, Austria; 2 Division of Hematology and Stem Cell Transplantation, Medical University of Graz, Graz, Austria; 3 Department of Blood Group Serology and Transfusion Medicine, Medical University of Graz, Graz, Austria; 4 Department of Blood Group Serology and Transfusion Medicine, Paracelsus Medical University, Salzburg, Austria; 5 Norwegian Center for Stem Cell Research, Institute of Basic Medical Sciences, University of Oslo, Oslo, Norway; 6 Cancer Biology Unit, Department of Dermatology, Medical University of Graz, Graz, Austria; 7 Department of Pediatric and Adolescence Surgery, Medical University of Graz, Graz, Austria; 8 Institute of Experimental and Clinical Cell Therapy, Paracelsus Medical University, Salzburg, Austria; Medical University Innsbruck, Austria

## Abstract

Therapeutic neo-vasculogenesis *in vivo* can be achieved by the co-transplantation of human endothelial colony-forming progenitor cells (ECFCs) with mesenchymal stem/progenitor cells (MSPCs). The underlying mechanism is not completely understood thus hampering the development of novel stem cell therapies. We hypothesized that proteomic profiling could be used to retrieve the *in vivo* signaling signature during the initial phase of human neo-vasculogenesis. ECFCs and MSPCs were therefore either transplanted alone or co-transplanted subcutaneously into immune deficient mice. Early cell signaling, occurring within the first 24 hours *in vivo*, was analyzed using antibody microarray proteomic profiling. Vessel formation and persistence were verified in parallel transplants for up to 24 weeks. Proteomic analysis revealed significant alteration of regulatory components including caspases, calcium/calmodulin-dependent protein kinase, DNA protein kinase, human ErbB2 receptor-tyrosine kinase as well as mitogen-activated protein kinases. Caspase-4 was selected from array results as one therapeutic candidate for targeting vascular network formation *in vitro* as well as modulating therapeutic vasculogenesis *in vivo*. As a proof-of-principle, caspase-4 and general caspase-blocking led to diminished endothelial network formation *in vitro* and significantly decreased vasculogenesis *in vivo*. Proteomic profiling *ex vivo* thus unraveled a signaling signature which can be used for target selection to modulate neo-vasculogenesis *in vivo*.

## Introduction

De-novo vessel formation is an essential step in organ regeneration as well as pathological manifestations such as ischemia and tumorigenesis which is mainly realized by sprouting angiogenesis in adult life [1].The formation of patent neo-vascular structures by stem/progenitor cells is termed neo-vasculogenesis and requires migration of transplanted or circulating endothelial lineage cells assembling the integral lining of newly formed vessels and mesenchymal cell-derived pericytes establishing and maintaining neo-vessel stability [1]–[3]. In embryonic development, vasculogenesis takes place in concert with rearrangement processes during which the formation of new vascular structures and regression of others occurs [4]. Vasculogenesis is presumably initiated through the crosstalk of cellular factors and secreted mediators, involving the regulation of various signaling pathways [5], [6]. We used an established neo-vasculogenesis model [7]–[9] to test our hypothesis of whether proteomic profiling can provide us with information on the *in vivo* signaling signature early during neo-vasculogenesis. The model was selected based on our hypothesis that human cell transplantation may allow for the recovery of a signaling signature derived from the transplanted cells. Anti-human antibodies used for array profiling were used to restrict the signature information to events related to transplanted cell-derived vasculogenesis. The rationale behind using the 80∶20 ratio was based on the previous observation that stable, perfused human vessels which connect to the murine circulation could be created in this model [9], [10]. We further tested whether the expressed signaling components can be targeted to influence vasculogenesis *in vivo*. Here we show that caspase-4 inhibition can block vasculogenesis *in vivo*, suggesting a role for caspase-4 activation in endothelial cell arrangement during vascular-like network formation *in vitro* as well as during experimental therapeutic neo-vasculogenesis *in vivo*.

## Materials and Methods

### Ethics Statement

Sample collection from human tissues was carried out after written informed consent from healthy donors according to procedures approved by the Ethical Committee of the Medical University of Graz (Protocols 19–252 ex 07/08, 18–243 ex 06/07, 21.060 ex 09/10, 19–252 ex 07/08). Adult samples were collected upon receiving written informed consent from healthy individuals. Umbilical cord (UC) and umbilical cord blood (UCB), placenta and amnion membrane samples were collected following full-term deliveries with written informed consent from the mothers according to the Declaration of Helsinki. All animal experiments were performed according to the 2010/63/EU guidance of the European Parliament on the welfare of laboratory animals. Protocols were given the approval of the Animal Care and Use Committee of the Veterinary University of Vienna on behalf of the Austrian Ministry of Science and Research.

### Cell Culture: Isolation and Characterization of ECFCs and MSPCs

**Figure 1:**
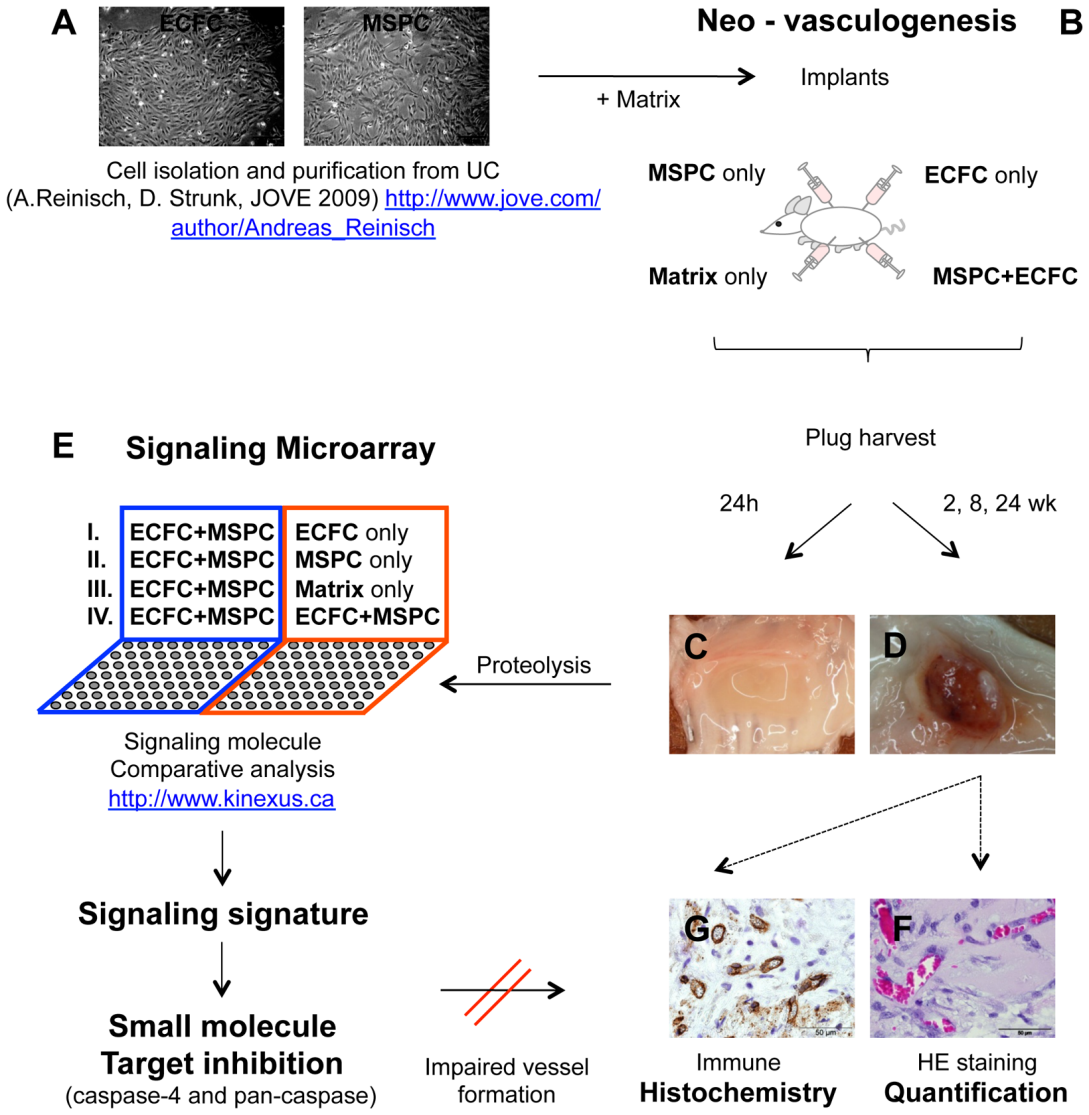
Overview of the project strategy. Autologous pairs of mesenchymal stem/progenitor cells (MSPC) and endothelial colony-forming progenitor cells (ECFC) were isolated, purified and expanded from the same human umbilical cord as described (http://www.jove.com/author/Andreas_Reinisch *)* (**A**)**.** Cells were combined with matrix and injected subcutaneously in four 300 µL implants per animal into the flank of immune compromised NSG mice as specified (**B**). Plugs were explanted 24 hours after implantation (**C**) to detect the signaling signature operative during the early phase of therapeutic vasculogenesis using proteomic profiling on customized antibody microarrays comparing either co-transplants of ECFC+MSPC with transplants containing sole ECFC, MSPC or Matrix *in vivo* (groups I – III), or co-transplants *in vivo* with a mixture of ECFC+MSPC created *in vitro* (group IV) (**E**)**.** Macroscopic view of plugs explanted after 2, 8 and 24 weeks (**C and D**). Histology and morphometry were performed to visualize vessel formation and stability in plugs in a time course (for up to 24 weeks) (**F and G**)**.** Small molecule inhibition of selected targets (from the array analysis) was used as a proof-of-principle confirming drugability of significantly regulated proteins within the early vasculogenesis signaling signature.

MSPC and ECFC isolation was performed as described before and illustrated in detail in a training video [11] (see also Figure 1 for the direct link). Briefly, cell expansion was performed using complete media supplemented with 10% pooled human platelet lysate (pHPL) replacing fetal bovine serum (FBS) as described in detail previously [2], [9], [12], [13]. ECFCs were isolated and expanded from human UC, UCB and white adipose tissue (WAT). MSPCs were isolated and expanded from UC, UCB and bone marrow (BM) [14], [15].

### Animal Experiments

Immune deficient NOD.Cg-Prkdc^scid^ Il2rg^tm1Wjl^/SzJ (NSG) mice were purchased from the Jackson laboratory (Bar Harbor, ME, USA), kept in the Medical University of Graz specific pathogen-free (SPF) facility and were used seven to 18 weeks after birth.

### *In vivo* Vessel Formation

ECFCs and MSPCs were isolated and purified as previously described [11] (see also Figure 1A). ECFCs were seeded in endothelial growth medium-2 (EGM-2, Lonza) at a density of 1,000 cells/cm^2^ and MSPCs in alpha-modified minimum essential medium (α-MEM, Sigma-Aldrich, St. Louis, MO) at a density of 500 cells/cm^2^ in 2,528 cm^2^ cell factories (CF-4, Thermo Fisher Scientific, Freemont, CA). Two million MSPCs (MSPC only), two million ECFCs (ECFC only) or the combination of 1.6×10^6^ ECFCs with 0.4×10^6^ MSPCs (ECFC+MSPC) were re-suspended in 300 µL ice-cold liquid extracellular matrix derived from the *In vitro* angiogenesis assay kit (Cat. No. ECM 625, Millipore, Billerica, MA, USA) and injected subcutaneously into NSG mice. Implants of cell-free ‘matrix only’ (Millipore) were used as controls (Figure 1B).

At days one, 14, 56 and 168 after implantation, mice were sacrificed by cervical dislocation and plugs were surgically removed from the subcutaneous sites (three mice and three plugs per combination per time point; Figure 1C and D). Three plugs harvested after 24 hours (day one samples) were used for generating protein lysates to detect early cell signaling molecules (Figure 1E), whereas parallel transplants were harvested at two and eight weeks (14 and 56 days, respectively) and were used for the histological confirmation of patent vessel formation in a time-course analysis (Figure 1F and G).

To evaluate the influence of caspase inhibition on *in vivo* vessel formation, either ECFCs or MSPCs or both cell types were pretreated with chemical caspase-4 inhibitor Z-LEVD-FMK (2 µM), pan-caspase inhibitor EZ-Solution™ Q-VD-OPh (10 µM; both BioVision Research Products, CA, USA) or vehicle (Dimethyl sulfoxide, DMSO, WakChemieMedical GmbH, Steinbach, Germany) for eight hours at 37°C prior to implantation (Figure 1). The cells were seeded in the 225 cm^2^ flasks, after reaching 70–80% confluence, were pretreated with caspase-4 and pan caspase inhibitors for 8 hours followed by 1× washing step with pre-warmed PBS (5 min, 300×g, 4°C). Viability of the cells was checked using trypan blue staining and cells were counted again before implantation.

### Antibody-mediated Detection of Signaling Molecules: Sample Preparation and Data Analysis

In order to detect signaling proteins, plugs containing a total number of 32×10^6^ cells (to allow for recovery of an appropriate protein amount based on previous titration) were explanted one day (24 h) after implantation (3 mice and 3 plugs per condition were used). Explants were homogenized using 400 µL Triton X-100 lysis buffer containing proteinase- and phosphatase-inhibitors (Roche, IN, USA) followed by magNAlyser centrifugation (700×g, 20 sec.), sonification (5×10 sec. with 10 sec. cooling steps in between; Imlab, Boutersem, Belgium) and ultra-centrifugation (100,000×g, 30 min; Beckman Coulter GmbH, Vienna, Austria). Protein concentrations were determined by a Bradford assay (Bio-Rad, CA, USA) and optical density (OD) was measured with a Spectramax instrument (Molecular Devices, Sunnyvale, CA, USA). Aliquots of 100 µg and 500 µg of the extracted protein were preserved at −80°C until further use. Protein lysates were subjected to the Kinex™ antibody microarray as a customized service (Kinexus Bioinformatics Corp., Vancouver, Canada, www.kinexus.ca) comparing different cellular compositions for protein expression alterations. Array results have been submitted to Gene Expression Omnibus (GEO accession N° GSE45896). Z normalization for the data was used to identify relevant regulated targets [16]. A Z-ratio of more than ±1.2 was considered to be a significant change based on the manufacturer’s instructions. A summary of the project strategy is shown in Figure 1.

Protein lysates were also subjected to western blot analysis for human caspase-4. Briefly, protein concentrations were measured with Bradford assay (BioRad). An amount of 15 µg/lane/protein lysate was run on a 10% SDS-polyacrylamide gel. The blot was then transferred to polyvinylidenedifluoride (PVDF) membrane and incubated with caspase-4 specific primary antibody (N-15, 0.1 µg/mL, Santa Cruz, CA) overnight compared to Glyceraldehyde 3-phosphate dehydrogenase (GAPDH, FL-335, 0.025 µg/mL, Santa Cruz) as house-keeping protein control followed by peroxidase-conjugated secondary antibodies incubation (donkey anti-goat, 0.025 µg/mL and goat anti-rabbit, 0.025 µg/mL, both Santa Cruz, for caspase-4 and GAPDH antibodies, respectively). To visualize the signals, the blot was exposed to ECL reagent (Amersham) and x-ray film. Signal intensity was measured using ImageJ software (http://rsbweb.nih.gov/ij/).

### Specific Cell Signaling Protein Inhibition in ECFCs and MSPCs

Small molecule caspase-4 and pan-caspase inhibitors were selected to representatively investigate the influence of significantly regulated caspases, displayed in the array, on vessel formation. For titration of the highest non-toxic inhibitory concentration of the selected protein inhibitors, ECFCs and MSPCs were seeded in triplicate at a density of 500 cells/cm^2^ and treated with serially diluted inhibitor concentrations (Z-LEVD-FMK from 0.2 µM to 5 µM, and Q-VD-OPh from 0.08 µM to 50 µM with 5-fold dilution steps) for up to 48 hours. The cells were treated with the appropriate amount of DMSO as a control (see Figure S6). To determine whether caspase inhibition affected the viability of ECFCs and MSPCs, we performed annexin V staining to evaluate the respective proportion of apoptotic cells by flow cytometry according to the manufacturer’s protocol (Navios cytometer, Beckman Coulter, www.bc-cytometry.com) and trypan blue exclusion to directly count and quantify viable and dead cells. Cell suspensions were washed once with phosphate-buffered saline (PBS) and centrifuged (5 min, 300×g, 4°C). Cell pellets were re-suspended in 100 µL ice-cold binding buffer and stained with 10 µL of annexin V. After a 15 min incubation in the dark, cells were analyzed on a FACS Calibur instrument (BD, Bioscience, CA, USA) within 30 min. Staurosporin-treated (2 µM; Tocriset™, Tocris bioscience, Bristol, UK) and DMSO-treated cells were used as positive and negative apoptosis controls, respectively. For trypan blue staining, trypsinized cells were incubated with 0.4% trypan blue solution (Gibco life technologies, Grand Island, NY, USA) using a Bürker-Türk counting chamber (Bio-Rad, CA, USA).

### *In vitro* Angiogenesis Assay

ECFCs (7.5×10^4^) were re-suspended in 2 mL EGM-2/10% pHPL and seeded on 9.2 cm^2^ polymerized matrix (Angiogenesis assay kit; Millipore, Billerica, MA, USA) as described [17]. To test the effect of small molecule caspase inhibitors on vascular-like network formation, ECFCs were pre-cultured with 2 µM of caspase-4 inhibitor [18] and 10 µM of pan-caspase inhibitor [19] for eight hours *in vitro*, washed with pre-warmed PBS once and seeded on the solidified matrix. After 24 hours, endothelial networks were documented with a Color View III camera on an Olympus IX51 microscope with the analySIS B acquisition software (all Olympus, Hamburg, Germany). Numbers of branching points were determined using ImageJ software (http://rsbweb.nih.gov) as described [10].

### Histological Analysis

Explanted plugs after 2, 8 and 24 weeks were fixed in 3.7% neutral-buffered formalin and dehydrated in a graded series of ethanol before embedded in paraffin. Sections (4 µm) were deparafinized in xylene, rehydrated in descending ethanol series and stained by a routine hematoxylin-eosin staining technique or immune histochemistry (IHC) as previously published [10]. Briefly, antigen retrieval procedures were performed at a high temperature at either pH = 6 or pH = 9 retrieval solution (70°C/160 W, 40 min) depending on the antibody’s properties. Additionally, H_2_O_2_ was used to block endogenous peroxidases (10 min) and Ultra V Block (Thermo Scientific, 5 min), mouse-on-mouse blocking (MOM, 1 h; Vector Laboratories, Burlingame, CA; USA) and serum-free protein block (30 min; Dako, Glostrup, Denmark) were used to minimize unspecific antibody binding. Slides were exposed to un-conjugated monoclonal mouse anti-human antibodies specifically binding vimentin (human mesodermal derived cell marker which can label mesenchymal and endothelial and hematopoietic cells, clone: V9, 0.78 µg/mL, Dako), CD31 (clone: JC70A, 5.15 µg/mL, Dako), and CD90 (clone: EPR3132, Abcam, Cambridge, MA, USA) for 30–60 min at room temperature (RT). Negative controls were processed identically, except that the primary antibodies were replaced by isotype-matched control antibodies in the appropriate concentration (IgG1, BD). The staining was developed with ultravision LP large-volume detection system horseradish peroxidase (HRP) polymer (Thermo Scientific) and diaminobenzidine (DAB) detection system (Vector) following the manufacturer’s protocol and counterstained with Mayer’s hematoxylin solution (Sigma-Aldrich).

To quantify vessel formation in ECFC+MSPC plugs, luminal structures containing red blood cells (RBCs) that were CD31 and vimentin positive were considered as perfused vessels. Although no CD31 staining is possible in MSPC-only plugs, the rare luminal structures containing red blood cells inside the plugs were considered to represent (presumably mouse-derived sprouting) vessels suitable for quantification. Two independent observers quantified RBC-containing luminal structures in five high power fields (200x original magnification) of hematoxylin-eosin (H&E)-stained sections from the related plugs using the counting function in ImageJ software. Additionally, sections from three different cutting depths (150 µm intervals) were examined.

### Statistics

All values are expressed as mean ± SD. Statistical differences were determined using unpaired student’s t-test. Differences were considered significant when the p-value was less than 0.05 (*), less than 0.001(**) or less than 0.0001(***).

## Results

### Neo-vasculogenesis*in vivo* Requires Contribution of ECFCs and MSPCs

**Figure 2:**
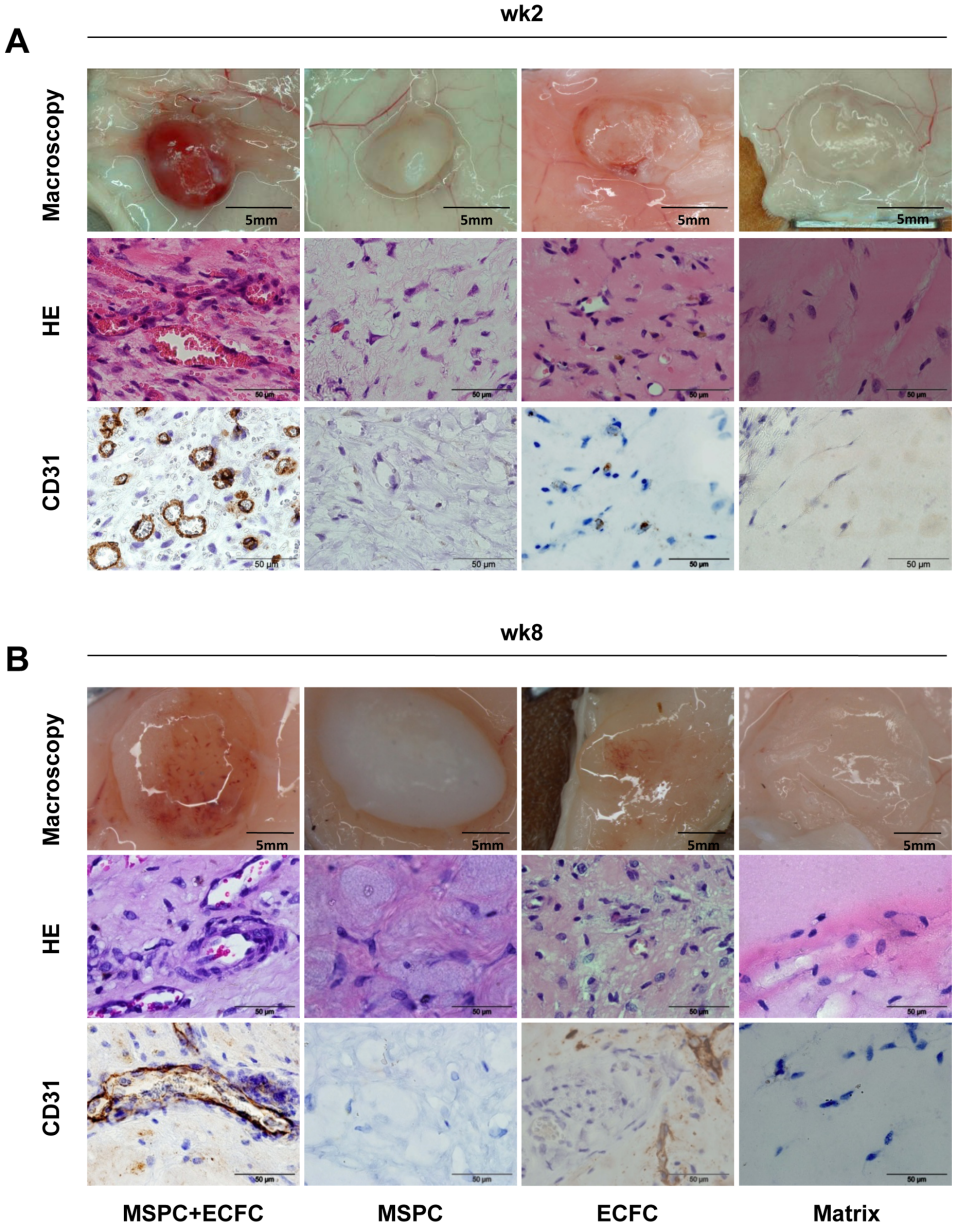
Macroscopic and microscopic features of the plugs two and eight weeks after implantation. Plugs were harvested two (**A**) and eight weeks (**B**) after subcutaneous co-transplantation of ECFC+MSPC (ratio 80∶ 20, left column), MSPC only (2^nd^ column), ECFC only (3^rd^ column), and cell-free matrix (right column). Representative macroscopic pictures of plugs in subcutaneous location (upper rows), micrographs of hematoxylin and eosin staining (HE, middle rows) and immune histochemistry combining human CD31 colored brown with hematoxylin counter-stain (huCD31, bottom rows) are depicted. Total cell number within the plugs was 2×10^6^ per 300 µL matrix (n = 3 per group and time course).

The phenotypic identity and purity of ECFCs and MSPCs was determined by flow cytometry (Figure S1). Subcutaneous implantation of ECFCs or MSPCs or co-transplantation of both cell types in an established 80∶20 ratio [9] was performed to study the mechanism by which these two UC-derived mesodermal cell types contribute to neo-vasculogenesis *in vivo* (Figure 1). Neo-vessel formation was evaluated macroscopically and histologically in the plugs harvested after two and eight weeks (3 mice and 3 plugs per condition, per time course were used; Figure 2). The red color of the harvested plugs after 2 and 8 weeks indicated perfusion which was confirmed by the presence of RBC-containing mature vessels after ECFC+MSPC co-transplantation (Figure 2A, B, upper panels). In accordance with previous data, immunohistochemistry verified the human origin of these newly formed vessels (Figure 2A, B, lower panels). The close interaction of ECFCs forming the luminal layer and MSPCs acting as pericytes stabilizing the vessel structure was also verified by human CD31 and CD90 analysis in the later time course (3 mice and 3 plugs per condition, per time course were used; Figure S2A). In contrast, the vessels in the implants that contained solely human MSPCs did not stain for human CD31, therefore presumably result from murine vessel in-growth (Figure 2A, B) as observed previously by others and us [10], [20]. A longer time course (8 weeks) of MSPC-only implantation *in vivo* results in an early stage of chondrogenesis (Figure 2B, middle panel), whereas a higher cell number and longer implantation time course led to vascularization in ECFC-only plugs *in vivo*. The plugs containing a combination of MSPC+ECFC *in vivo* became vascularized after 2 weeks, indicating a supportive role for MSPCs acting as pericytes to stabilize the established micro-vessels as shown previously [9]. The long term persistence and functionality of human co-transplant-derived vessels *in vivo* was verified for up to 24 weeks in plugs harvested after 168 days using anti-human vimentin immune histological analysis. The use of human anti-vimentin IHC in this study was based on the fact that ECFCs and MSPCs are mesodermal-derived cells and therefore known to express vimentin as a surface marker (3 mice and 3 plugs per condition per time course were used). These human-derived vessels remained stable in the plugs regardless of the original implanted cell density (Figure S2B). The number of injected and harvested plugs was three per condition and time point.

### ECFC and MSPC Signaling Signature after Co-transplantation

The use of ECFCs and MSPCs from the same umbilical cord (autologous cell pairs) in this study was done intentionally to exclude histocompatibility antigen-dependent protein display during array analysis or inter-individual variation when using cells derived from different donors. In order to detect the early signaling signature between ECFCs and MSPCs during neo-vasculogenesis, we analyzed the signaling molecules in lysates derived from plugs harvested 24 hours after subcutaneous implantation in advance of vessel assembly (Figure 1C; 3 mice and 3 plugs per condition per time course were used). The array results have been submitted to Gene Expression Omnibus (accession number GSE45896). Differences in signaling protein expression as well as phosphorylation status were tested comparing lysates derived from transplantation of ECFCs only or MSPCs with co-transplants (3 plugs per condition were used; Figure 1E). The communication between both cell types resulted in successful vasculogenesis as evidenced in parallel control transplants (Figure 1D, F, G and Figure 2).

**Figure 3:**
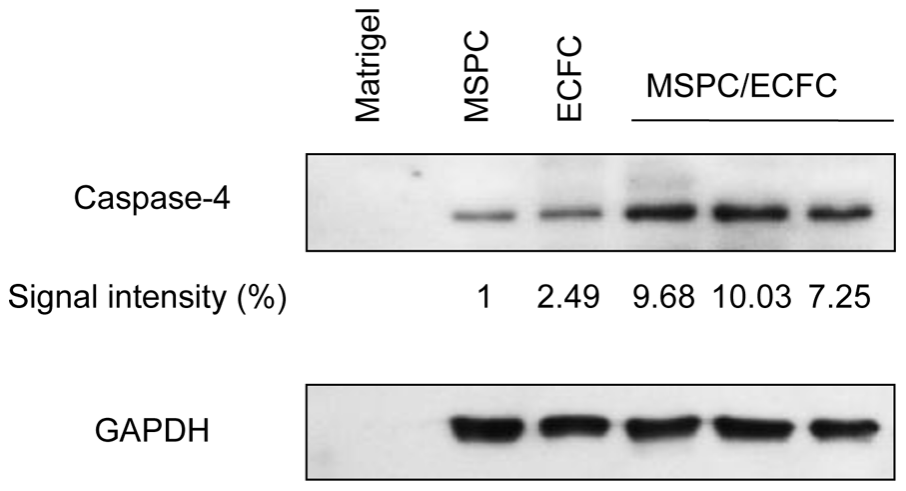
Western blot analysis of protein lysates from early transplants. Expression of human caspase-4 in MSPC, ECFC and co-transplants of the two cell populations as indicated was analyzed in equal amounts of protein lysates obtained from plugs harvested 24h after transplantation. A higher relative caspase-4 expression in MSPC/ECFC co-transplants as compared to the sole populations is illustrated (top graph) and verified using western blot quantification with ImageJ software compared to the GAPDH signal. The quantification values (Caspase-4/GAPDH area) were measured and the MSPC only signal set as one. Complete blot scans are shown in Figure S5.

Protein array analysis revealed that most of the significant (Z-ratio equal to or more than ±1.2) differentially regulated signaling molecules in neo-vasculogenesis-competent co-transplants did belong either to the kinase or caspase families. Caspase-4 and -7 were significantly up-regulated in 4/4, caspase-2 and -12 in 3/4 and caspase-5 and -6 in 1/4 co-transplant microarrays when compared to ECFC (group I) or MSPC transplants (group II), empty matrix (group III) or an *in vitro* ECFC/MSPC mixture (group IV). DNA protein kinase (DNAPK), calcium/calmadulin-dependent protein kinase-2 (CaMK-2), never-in-mitosis-related protein-serine kinase 2 (Nek2), and the human ErbB2 receptor-tyrosine kinase were expressed with higher frequency in 4/4 and 3/4 co-transplant microarrays, respectively. Extracellular signal-regulated serine protein kinase (Erk 1/2) was up-regulated in 2/4 co-transplant microarrays. The FLJ35932 serine kinase (STK33), serine phosphatase 1 catalytic subunit (PP1/Cb) and nerve growth factor receptor tyrosine kinase (NTRK1) were down-regulated in four, three and two comparative arrays, respectively (Table 1). Up-regulation of caspase-4 protein in ECFC/MSPC co-transplants 24 h after transplantation was further confirmed by western blot analysis of the protein lysates derived from the same explants that were subjected to protein array analysis (Figure 3).

**Table 1:** Signaling signature specific for effective ECFC+MSPC co-transplantation in antibody microarrays.

ECFC+MSPC	I. ECFC	II. MSPC	III. Matrix	IV. in vitro
CASP 2	↑ 1.38	↑ 2.06	↑ 1.29	
CASP 4	↑ 1.73	↑ 2.58	↑ 1.57	↑ 1.47
CASP 5		↑ 1.80		
CASP 6	↑ 1.78			
CASP 7	↑ 2.18	↑ 2.61	↑ 1.86	↑ 1.27
CASP 12	↑ 2.19		↑ 2.06	↑ 1.99
DAXX	↑ 1.87	↑ 2.84		
CAMK 2α	↑ 1.49	↑ 2.40	↑ 1.51	
CAMK 2β	↑ 1.97	↑ 2.73	↑ 1.65	↑ 1.34
CAMK 2γ	↑ 1.83	↑ 2.48	↑ 1.53	
CAMK 2δ			↑ 1.77	↑ 1.25
DNAPK	↑ 1.37	↑ 1.97	↑ 1.41	↑ 1.22
PACSIN 1	↑ 1.51			↑ 1.43
DGKz	↑ 1.87	↑ 2.17		↑ 1.22
TTK		↓ 2.12		↓ 1.25
Erk 1/2		↑ 1.34	↑ 1.21	
Nek 2	↑ 1.45		↑ 1.32	↑ 1.49
PP1/Cb	↓ 1.94	↓ 2.86		↓ 1.65
STK 33	↓ 2.30	↓ 2.53	↓ 1.90	↓ 1.21
ErbB 2	↑ 1.99	↑ 2.75	↑ 1.82	
NTRK 1(TrkA)	↓ 2.00	↓ 1.72		

Signaling molecules expressed significantly in co-transplants of ECFC+ MSPC *in vivo* compared to that of equal numbers of ECFC only, MSPC only, Matrix only without cells [all harvested 24 h after transplantation] and ECFC+MSPC mixture *in vitro* are shown. The total number of microarrays is four with each target antibody determined in duplicate (1,760 spots per condition). ↑ arrows indicate up-regulation and ↓ arrows down-regulation of the target in ECFC+MSPC *in vivo* compared to the four different groups. Z-ratio of more than ±1.2 was considered to be significant. The results have been submitted to Gene Expression Omnibus (accession number GSE45896). For a complementary list of the expressed signaling molecules see the Table S1.

**Casp4:** Caspase 4 (ICH2 protease, ICE(rel)-II); **Casp7:** Caspase 7 (ICE-like apoptotic protease 3 (ICE-LAP3), Mch3); **CAMK2b:** Calcium/calmodulin-dependent protein-serine kinase 2 beta; **DNAPK:** DNA-dependent protein kinase catalytic subunit; **Casp2:** Caspase 2 (ICH1 protease); **Casp12:** Caspase 12 (mouse); **CAMK2g:** Calcium/calmodulin-dependent protein-serine kinase 2 gamma; **ErbB2:** ErbB2 (Neu) receptor-tyrosine kinase; **DGKz:** Diacylglycerol kinase zeta; **Nek2:** NIMA (never-in-mitosis)-related protein-serine kinase 2; **Hsp27:** Heat shock 27 kDa protein beta 1 (HspB1); **DAXX:** Death-associated protein 6 (BING2); **HO-1:** Hemeoxygenase 1; **CAMK2a:** Calcium/calmodulin-dependent protein-serine kinase 2 alpha; **Casp5:** Caspase 5 (ICH3 protease, ICE(rel)-III); **Casp6:** Caspase 6 (apoptotic protease Mch2); **PACSIN1:** Protein kinase C+casein kinase substrate in neurons protein 1; **Erk 1/2:** Extracellular regulated protein-serine kinase 1; **CAMK2d:** Calcium/calmodulin-dependent protein-serine kinase 2 delta; **FAK:** Focal adhesion protein-tyrosine kinase; **FGFR1:** Fibroblast growth factor receptor-tyrosine kinase 1; **EphA1:** Ephrin type-A receptor 1 protein-tyrosine kinase; **P53:** Tumor suppressor protein p53 (antigenNY-CO-13).

### Blocking Caspase Signaling Results in Deficient ECFC Network Formation*in vitro*

**Figure 4:**
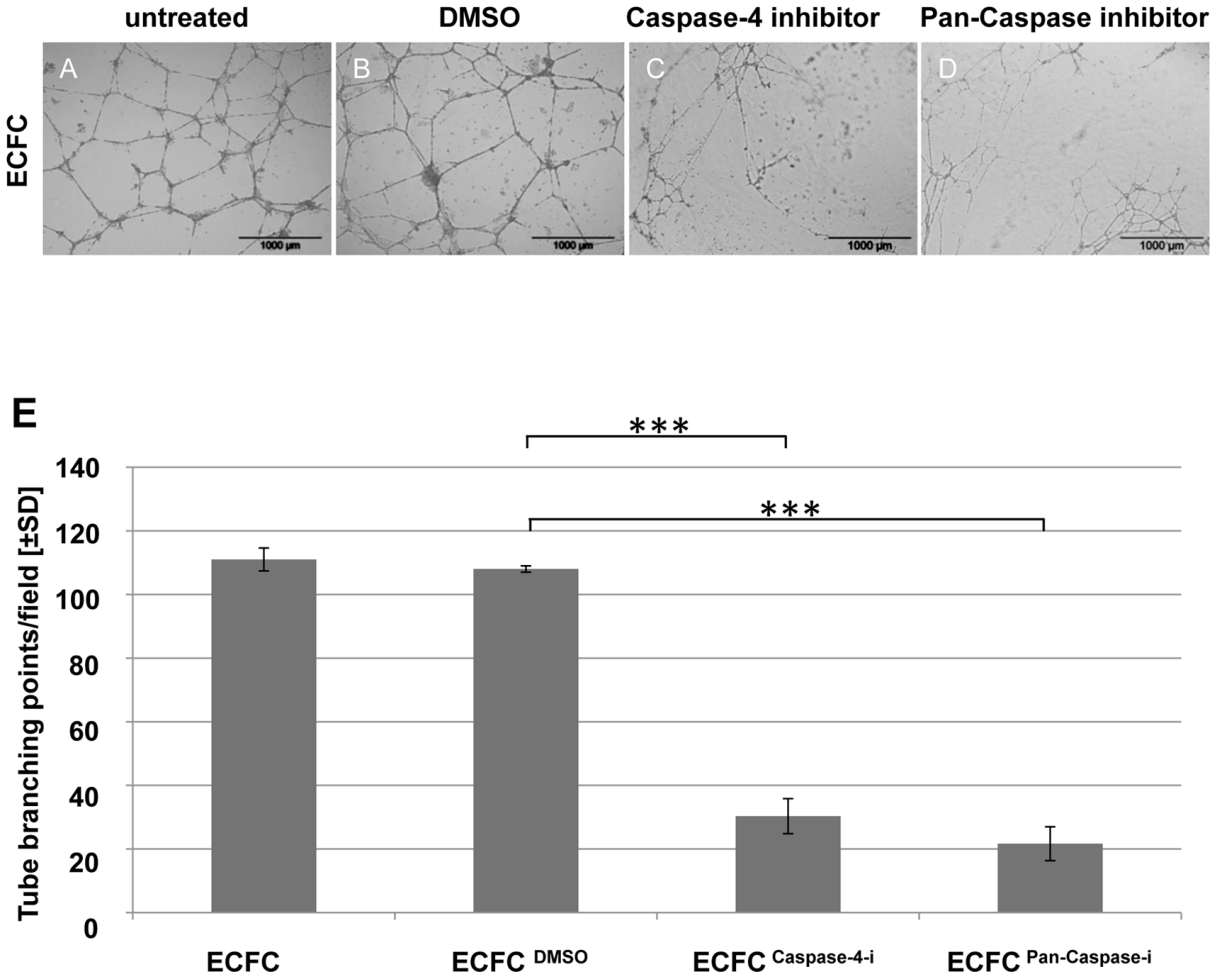
Effect of caspase inhibitors on ECFC network formation***in vitro*** **.** ECFCs were tested un-treated (**A**) and upon eight hour pretreatment with either DMSO (**B**), Caspase-4 inhibitor Z-LEVD-FMK (**C**) or pan-caspase inhibitor Q-VD-OPh (**D**) in a 24 hour angiogenesis assay *in vitro*. Quantification of cell network branching points and corresponding un-paired t-test results (*** p≤0.0001) are depicted (**E**). Each field represents an area of 9.45 mm^2^. Scale bar: 1000 µm, n = 3 per condition.

Vascular-like network formation represents a frequently used surrogate model for studying vasculogenesis *in vitro* [17], [21], [22]. We analyzed the effect of caspase-4 and general caspase inhibition on *in vitro* network formation as an indicator of the relevance of the selected signaling signature for *in vivo* vessel formation. To avoid toxic side effects, concentrations of caspase-4 and pan-caspase inhibitors were titrated based on previous studies [18], [19], [23] (Figure S6). Untreated UC-derived ECFCs capable of forming vascular-like networks covering the matrix area after 24 hours were used as a positive control (Figure 4A). Pretreatment with caspase-4 and pan-caspase inhibitor significantly inhibited network formation. ECFC network quantification revealed a considerably reduced number of branching points in caspase-4 and pan-caspase inhibitor treated cells. Network formation and number of branching points remained unaffected when only the solvent (DMSO) was used for pretreatment of the ECFCs (Figure 4, B-E). The experiment was performed using three different UC-ECFC donors.

### Blocking Caspase Signaling Impairs Neo-vasculogenesis*in vivo*

Since the co-transplantation of ECFCs together with MSPCs reproducibly resulted in the generation of perfused human vessels *in vivo,* this model was used to evaluate the effect of targeting caspase signaling during early neo-vessel formation. We found that plugs containing UC-derived ECFCs plus MSPCs pretreated either with caspase-4 or pan-caspase inhibitor and harvested after two weeks of implantation, showed reduced vasculogenesis (2 mice and 2 plugs per condition were used; Figure S3A).

To exclude the possibility that the role of caspases was restricted to UC-derived cells undergoing neo-vasculogenesis, other cell sources were additionally assessed. Despite variation in the absolute numbers of vessels created, the caspase inhibition impaired vessel formation irrespective of the ECFC/MSPC sources combined (Figure S3; 2 mice and 2 plugs per condition were used).

**Figure 5:**
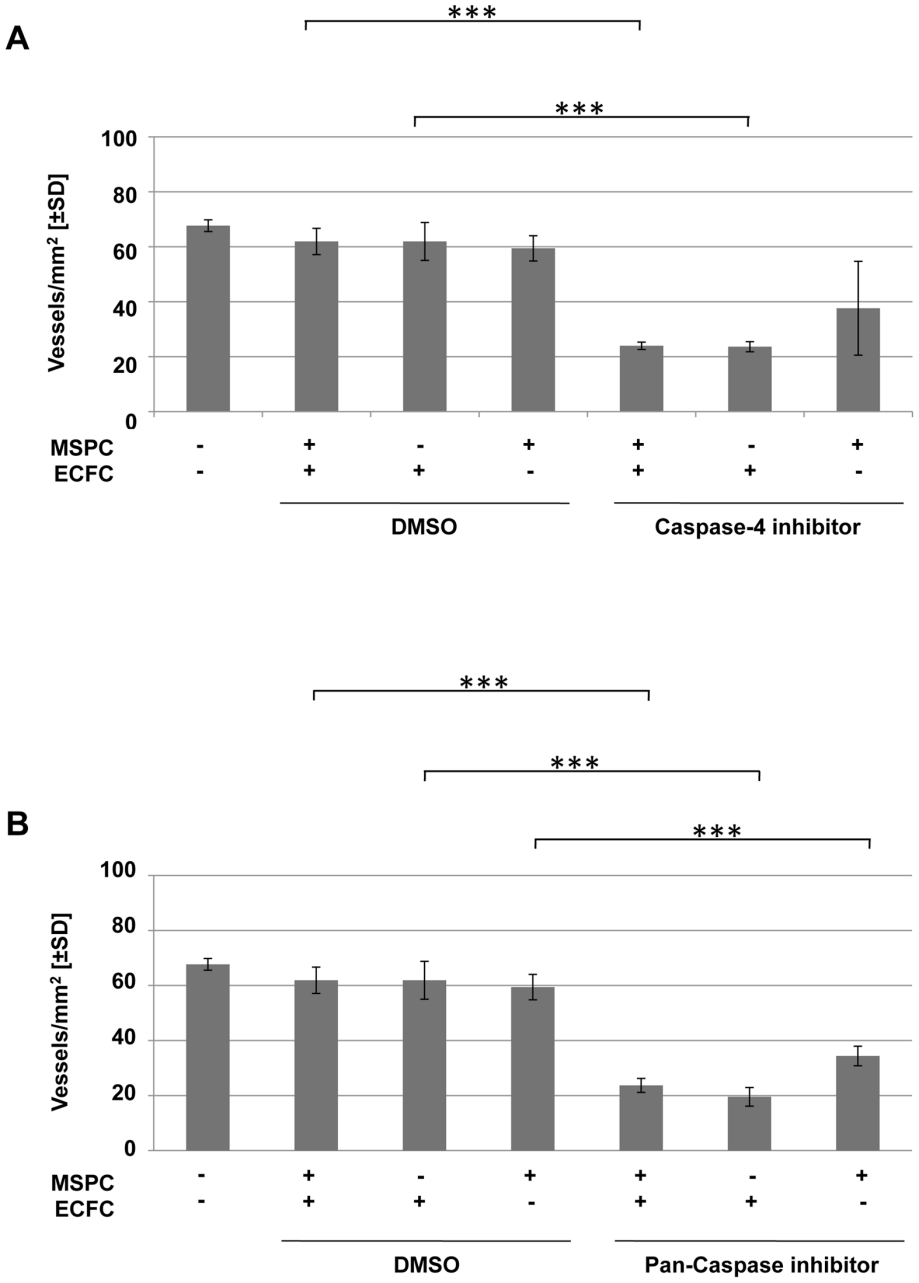
Vessel formation is impaired upon caspase-4 and pan-caspase inhibition. Mean number of vessels created by ECFC+MSPC co-transplantation of MSPC+ECFC pairs derived from three different human organs (for primary data see Figures S3 and S4: umbilical cord-derived ECFC+umbilical cord-derived MSPC; white adipose tissue-derived ECFC+umbilical cord blood-derived MSPC; umbilical cord blood-derived ECFC+bone marrow-derived MSPC) comparing untreated progenitor cells with those pretreated either with DMSO **(A and B, left panels),** Caspase-4 inhibitor **(A, right panels)** or pan-caspase inhibitor **(B, right panel)** (** p<0.001, *** p≤0.0001; n = 6 mice and 6 plugs per pretreatment; regardless of the cell source and combination).

Irrespective of the ECFC source, a significant reduction of vessel formation was observed when ECFCs were treated by a caspase-4 blocker before co-transplantation (6 mice and 6 plugs per condition were used). Whereas caspase-4 blocking of MSPCs did not have a significant impact on vasculogenesis, blocking both cell types led to impaired vasculogenesis comparable to ECFC blocking only. These findings were again irrespective of the ECFC or MSPC source used (Figure 5A). In contrast, neo-vasculogenesis was significantly impaired upon pan-caspase inhibition of either cell type alone or the combination (two plugs per pretreatment per cell source; Figure 5B).

To eliminate a possible bias due to uneven distribution of vessel formation within the three-dimensional transplant space, vascular structures were additionally quantified in three different depths of sections within the plugs harvested after two weeks. Despite certain variation in the number of vessels in different depths, vessel density did not differ significantly in either untreated samples or upon caspase-4 or pan caspase inhibitor pretreatment of either or both cell types in all three cell compositions (Figure S4).

## Discussion

The aim of this study was to identify the signaling pathways which are operative in the early phase of vasculogenesis after co-transplantation of ECFCs and MSPCs *in vivo*. The aim was accomplished by (1) defining 57 significantly regulated signaling molecules by antibody array proteomic profiling of 24-h-explants of ECFC+MSPC co-transplants and (2) illustrating the impact of caspase-4 inhibition as one prototypic pathway in neo-vessel formation through the component’s inhibition as a proof-of-principle.

We used a proteomic profiling strategy with antibody-mediated detection of signaling molecules to identify the specific signaling signature in early co-transplants. The comparative strategy allowed us to recognize signals that are activated after co-transplantation as compared to transplants of either single cell population. Protein expression analysis in extracellular matrix without admixed cells helped to subtract background signaling. In a fourth group we compared the supposed initial vasculogenesis situation (24h after ECFC+MSPC co-transplantation) with just an *in vitro* mixture of ECFCs+MSPCs (to exclude signals derived from a mixture of the two progenitor cell types in the absence of vasculogenesis). Our protein microarray data analysis revealed significant induction of various caspases and the death-associated protein 6 (DAXX). Apoptosis signaling is a crucial step for tissue repair and remodeling and is considered to be a survival mechanism [24]. Disturbance of apoptosis mechanisms can result in defective angiogenesis as reported previously [25]. The role of death-associated pathways through caspase activation during the initiation of vessel formation or radiation sensitization of solid tumors using caspase inhibition has been studied in different angiogenesis and tumor models [25], [26]. The pan-caspase inhibitor Z-VAD and selective inhibitors of caspase-3 and caspase-8 have been shown to increase radition sensitivity resulting in tumor growth delay *in vivo* [26], [27]. It has also been shown that inhibition of executioner caspase-8 reduced adhesion and migration of endothelial progenitor cells (EPCs) and their capacity to enhance neo-vasculogenesis [28]. Contradictory earlier reports showed that activation of apoptotic pathways can block vessel formation [29], [30]. Despite this ongoing controversy regarding the role of apoptosis during angiogenesis, the precise role of caspases and particularly caspase-4 during therapeutic vasculogenesis has yet to be studied.

Human caspase-4 is a poorly characterized member of the caspase-1 subfamily localized in the endoplasmic reticulum (ER) membrane [31]–[33]. However, its role in activation of inflammosomes via caspase-1 regulation [34] as well as involvement in ER stress-induced apoptosis has been demonstrated [18], [31]. In our study, caspase-4 was significantly expressed with higher abundance in all four co-transplant microarrays, which suggests an activation of this inflammatory caspase in the early stage of neo-vasculogenesis, together with other initiator and executioner caspases such as caspase-2, -5, -6 and -7. The broad spectrum pan-caspase inhibitor also used in this study [19] blocks executioner (caspase-3) [35], initiator (caspase-8, -9, -10) [36] and inflammatory caspase-12 [37]. Since the role of initiator caspase-8 and executioner caspase-3 and caspase-7 for survival of endothelial cells and during angiogenesis *in vivo* has been demonstrated previously [25]–[28], we conclude that these caspases are of comparable importance also during vasculogenesis. Our data also established the as of yet unknown role of the inflammatory caspase-4 in regulating neo-vasculogenesis. We used small molecule caspase-4 (Z-FMK-LEVD) and, as a positive control, pan-caspase (Q-VD-OPh) inhibitors that irreversibly block caspases without cyto-toxicity effect [18], [19], [23], thus verifying the drugability of caspases as targets in this experimental therapeutic application. Whether caspase-4 plays a progenitor cell-specific role in apoptosis-induced proliferation during tissue regeneration remains to be studied [38]. Several protein kinases are also known to be involved in vessel formation. CaMKs are main mediators of calcium signaling during proliferation, motility and development of eukaryotic cells [39]. CaMKs are also involved in tumor progression via the regulation of a variety of cellular signaling pathways [40], [41]. They can activate the hypoxia-inducible transcription factor (HIF-1) resulting in vascular endothelial growth factor (VEGF) expression [42]. We have recently demonstrated that MSPCs rescue ECFCs from hypoxia-induced apoptosis during therapeutic vasculogenesis by a HIF-1-dependent mechanism [10]. In the current study we also detected the up-regulation of inflammatory caspase-12 in the majority (three of four) of neo-vasculogenesis arrays, which may be additional evidence of the involvement of inflammatory caspases in neo-vasculogenesis. The precise mechanism of integrating caspase activity and hypoxia sensing in a functional crosstalk between ECFCs and MSPCs during effective vasculogenesis will be addressed in future studies.

Focal adhesion tyrosine kinase (FAK) is involved in early integrin signaling and has been shown to reduce vascular structure formation upon down-regulation in glioma cells. Moreover, down-regulation of FAK, VEGF and protein kinase B (Akt) inhibited vessel formation in human UCB-treated nude mice [5]. Detection of CaMK and FAK up-regulation during efficient neo-vasculogenesis in this study confirms the relevance of the employed strategy. Unraveling the non-apoptotic side of caspase components during vasculogenesis crosstalk will help us to better understand the concerted interplay of the different stem/progenitor populations during tissue regeneration and will hopefully contribute to developing new therapeutic approaches. In addition we have established a platform for evaluating the efficiency of other drugs including kinase inhibitors during vascular regeneration. One drawback of the current study is that we are not able to define whether the initial caspase-4 signal arose from ECFCs or MSPCs in the co-transplant protein lysate. A subtractive approach using pre-treatment of one or the other cell population to inhibit caspase-4 in ECFCs or MSPCs may help to determine whether both or just one of the two cell populations utilize caspase-4 during efficient vasculogenesis. Preliminary results (Figure 3 and Figure S3) may indicate a particular role of caspases in the ECFC compartment. The significantly regulated molecules within the displayed signaling signature of early therapeutic vasculogenesis represent attractive targets for therapeutic intervention in this model. We are well aware of the fact that the relatively ischemic plugs in this model situation do not represent true organ damage and signals arising from damaged tissues may have additional effects on the cells during vascular repair. Further analysis in additional models for angiogenesis and vasculogenesis is required to determine which of the selected molecules can be targeted efficiently to modulate vessel formation.

### Conclusions

In this study, we analyzed the crosstalk between ECFCs and MSPCs during neo-vasculogenesis, which regulates the formation of functional and long-term stable vessels. We identified significantly expressed mediators including caspase signaling through which the early crosstalk takes place. Our hypothesis that an effective signaling signature during early vasculogenesis can be detected by *ex vivo* proteomic profiling was verified by showing impaired network and vessel formation by blocking caspase components *in vitro* and *in viv*o, respectively. These results led us to speculate that inflammatory caspase-4 has a role in the initiation of vessel formation by ECFC+MSPC co-transplantation. A better understanding of the mechanism underlying neo-vasculogenesis will help to develop rational therapeutic strategies.

## Supporting Information

Figure S1 **Immune phenotype of culture-expanded cells.** Culture-expanded mesenchymal stem/progenitor cells (MSPC; upper rows) and endothelial colony-forming progenitor cells (ECFC; lower rows) as used for co-transplantation were analyzed by flow cytometry for their immune phenotype. Red lines indicate target antibody and blue lines isotype control reactivity. All MSPC used were >95% positive for CD90, CD73 and CD105 fulfilling common ISSCR criteria and lacked reactivity with hematopoietic markers (<2%) as exemplified by CD15 and CD19. ECFC were completely CD90-negative excluding MSPC contamination and their purity was confirmed by CD31 reactivity. One representative example is shown.
(TIF)

Figure S2 **Human vessel formation and persistence.** (**A**) Perfused vessels within ECFC+MSPC co-transplants harvested after two (wk2, left column) and eight weeks (wk8, right column) were visualized by anti-human CD31 (upper row, CD31, brown) and anti-human CD90 immune histochemistry (middle row, brown), verifying contribution of ECFCs to vascular lumen and presence of MSPCs around capillaries. Human CD31/CD90 double staining (lower row, CD31^+^ in brown, CD90^+^ in red) depicts intimate localization of MSPCs to ECFCs in vascular structures. Nuclei were counterstained with hematoxylin (blue). (**B**) Plugs containing umbilical cord derived ECFC+MSPC with a total cell number of 2×10^6^ (left column, n = 2), 8×10^6^ (middle, n = 2) and 32×10^6^ (the latter representing the cell number utilized for obtaining sufficient amounts of protein for antibody array analysis in the initial experiments; right column, n = 2) were harvested 24 weeks after implantation and stained with anti-human vimentin (brown); nuclei are counterstained in blue by hematoxylin.
(TIF)

Figure S3 **Caspase inhibition hampers human progenitor cell-derived vasculogenesis** ***in vivo.*** Vascular structure quantification upon DMSO (left panel), caspase-4 (middle panel) and pan-caspase pre-treatment of either MSPC or ECFC or both cell types compared to untreated conditions are shown. (**A**) Autologous (to each other) MSPC and ECFC were derived from term umbilical cord and culture-expanded before transplantation mimicking the conditions used for the antibody array analysis (n = 2 per condition). (**B**) White adipose tissue-derived ECFC were co-transplanted with allogeneic umbilical cord blood-derived MSPC to exclude that the inhibitory effect of caspases in this process is restricted to cord-derived cells or specific for autologous progenitor cell pairs (n = 2 per condition). (**C**) Umbilical cord blood-derived ECFC were co-transplanted with bone marrow-derived MSPC confirming that the inhibitory effect of caspases in this process is not restricted to cord-derived cells and not specific for autologous progenitor cell pairs (n = 2 per condition).
(TIF)

Figure S4 **Quantification of vessel density upon caspase inhibition in various depths of the implants.** Plugs containing human vessels created by co-transplantation of umbilical cord (UC)-derived ECFC+UC-MSPC (left panels); white adipose tissue (WAT)-derived ECFC+umbilical cord blood (UCB)-derived MSPC (middle panels) and UCB-ECFC+bone marrow (BM)-derived MSPC (right panels). Progenitor cells were either pre-treated (+) or left un-treated (−) with vehicle DMSO or (**A**) caspase-4 or (**B**) pan-caspase inhibitor as indicated. Explanted (after 2 weeks) and fixed plugs were cut in three depths with approximately 150 µm intervals and the micro-vessels were counted in different sections. This strategy was used to minimize a hypothetic uneven vessel distribution bias in micro-vessel quantifications within plugs.
(TIF)

Figure S5 **Western blot scan of Caspase-4 and GAPDH analysis.** Western blots of Matrix, MSPCs, ECFCs and three independent co-transplants of MSPCs/ECFCs using the protein lysates of the plugs 24 h after transplantation. The Blot was incubated with anti-human caspase-4 (**A**) or GAPDH antibodies (**B**). Uncut membrane of the representative results is shown in figure 3.
(TIF)

Figure S6 **Limited cytotoxicity of caspase inhibitors.** MSPC and ECFC were subjected to control (DMSO) or increasing concentration of DMSO-dissolved (**A**) caspase-4 or (**B**) pan-caspase inhibitor pre-treatment for 8 h. Cells were washed 2x and Annexin-V-reactivity was determined by flow cytometry to measure apoptotic and dead cells. Percentages of viable annexin negative cells are depicted (mean ± SD of three experiments).
(TIF)

Table S1 **Complementary list of signaling molecules expressed in antibody microarrays.**
(DOCX)

## References

[1] Carmeliet P, Jain RK (2011) Molecular mechanisms and clinical applications of angiogenesis. Nature 473: 298–307. 21593862 10.1038/nature10144 PMC4049445

[2] Reinisch A, Bartmann C, Rohde E, Schallmoser K, Bjelic-Radisic V, et al (2007) Humanized system to propagate cord blood-derived multipotent mesenchymal stromal cells for clinical application. Regen Med 2: 371–382. 17635045 10.2217/17460751.2.4.371

[3] Losordo DW, Dimmeler S (2004) Therapeutic angiogenesis and vasculogenesis for ischemic disease. Part I: angiogenic cytokines. Circulation 109: 2487–2491. 15173038 10.1161/01.CIR.0000128595.79378.FA

[4] Risau W, Flamme I (1995) Vasculogenesis. Annu Rev Cell Dev Biol 11: 73–91. 8689573 10.1146/annurev.cb.11.110195.000445

[5] Dasari VR, Kaur K, Velpula KK, Dinh DH, Tsung AJ, et al (2010) Downregulation of Focal Adhesion Kinase (FAK) by cord blood stem cells inhibits angiogenesis in glioblastoma. Aging (Albany NY) 2: 791–803. 21068464 10.18632/aging.100217 PMC3006022

[6] Dumont DJ, Yamaguchi TP, Conlon RA, Rossant J, Breitman ML (1992) tek, a novel tyrosine kinase gene located on mouse chromosome 4, is expressed in endothelial cells and their presumptive precursors. Oncogene 7: 1471–1480. 1630810

[7] Au P, Daheron LM, Duda DG, Cohen KS, Tyrrell JA, et al (2008) Differential in vivo potential of endothelial progenitor cells from human umbilical cord blood and adult peripheral blood to form functional long-lasting vessels. Blood 111: 1302–1305. 17993613 10.1182/blood-2007-06-094318 PMC2214740

[8] Mead LE, Prater D, Yoder MC, Ingram DA (2008) Isolation and characterization of endothelial progenitor cells from human blood. Curr Protoc Stem Cell Biol Chapter 2: Unit 2C 1. 10.1002/9780470151808.sc02c01s6 18770637

[9] Reinisch A, Hofmann NA, Obenauf AC, Kashofer K, Rohde E, et al (2009) Humanized large-scale expanded endothelial colony-forming cells function in vitro and in vivo. Blood 113: 6716–6725. 19321860 10.1182/blood-2008-09-181362 PMC2710924

[10] Hofmann NA, Ortner A, Jacamo RO, Reinisch A, Schallmoser K, et al (2012) Oxygen sensing mesenchymal progenitors promote neo-vasculogenesis in a humanized mouse model in vivo. PLoS One 7: e44468. 22970226 10.1371/journal.pone.0044468 PMC3436890

[11] Reinisch A, Strunk D (2009) Isolation and animal serum free expansion of human umbilical cord derived mesenchymal stromal cells (MSCs) and endothelial colony forming progenitor cells (ECFCs). J Vis Exp. 10.3791/1525 PMC3164069 19816400

[12] Schallmoser K, Bartmann C, Rohde E, Reinisch A, Kashofer K, et al (2007) Human platelet lysate can replace fetal bovine serum for clinical-scale expansion of functional mesenchymal stromal cells. Transfusion 47: 1436–1446. 17655588 10.1111/j.1537-2995.2007.01220.x

[13] Hofmann NA, Reinisch A, Strunk D (2012) Endothelial colony-forming progenitor cell isolation and expansion. Methods Mol Biol 879: 381–387. 22610572 10.1007/978-1-61779-815-3_23

[14] Shahdadfar A, Fronsdal K, Haug T, Reinholt FP, Brinchmann JE (2005) In vitro expansion of human mesenchymal stem cells: choice of serum is a determinant of cell proliferation, differentiation, gene expression, and transcriptome stability. Stem Cells 23: 1357–1366. 16081661 10.1634/stemcells.2005-0094

[15] Szoke K, Beckstrom KJ, Brinchmann JE (2012) Human adipose tissue as a source of cells with angiogenic potential. Cell Transplant 21: 235–250. 21669039 10.3727/096368911X580518

[16] Cheadle C, Vawter MP, Freed WJ, Becker KG (2003) Analysis of microarray data using Z score transformation. J Mol Diagn 5: 73–81. 12707371 10.1016/S1525-1578(10)60455-2 PMC1907322

[17] Rohde E, Bartmann C, Schallmoser K, Reinisch A, Lanzer G, et al (2007) Immune cells mimic the morphology of endothelial progenitor colonies in vitro. Stem Cells 25: 1746–1752. 17395771 10.1634/stemcells.2006-0833

[18] Bian ZM, Elner SG, Elner VM (2009) Dual involvement of caspase-4 in inflammatory and ER stress-induced apoptotic responses in human retinal pigment epithelial cells. Invest Ophthalmol Vis Sci 50: 6006–6014. 19643964 10.1167/iovs.09-3628 PMC3208232

[19] Caserta TM, Smith AN, Gultice AD, Reedy MA, Brown TL (2003) Q-VD-OPh, a broad spectrum caspase inhibitor with potent antiapoptotic properties. Apoptosis 8: 345–352. 12815277 10.1023/a:1024116916932

[20] Allen P, Melero-Martin J, Bischoff J (2011) Type I collagen, fibrin and PuraMatrix matrices provide permissive environments for human endothelial and mesenchymal progenitor cells to form neovascular networks. J Tissue Eng Regen Med 5: e74–86. 21413157 10.1002/term.389 PMC3178449

[21] Grant DS, Tashiro K, Segui-Real B, Yamada Y, Martin GR, et al (1989) Two different laminin domains mediate the differentiation of human endothelial cells into capillary-like structures in vitro. Cell 58: 933–943. 2528412 10.1016/0092-8674(89)90945-8

[22] Davis GE, Camarillo CW (1995) Regulation of endothelial cell morphogenesis by integrins, mechanical forces, and matrix guidance pathways. Exp Cell Res 216: 113–123. 7813611 10.1006/excr.1995.1015

[23] Kuzelova K, Grebenova D, Brodska B (2011) Dose-dependent effects of the caspase inhibitor Q-VD-OPh on different apoptosis-related processes. J Cell Biochem 112: 3334–3342. 21751237 10.1002/jcb.23263

[24] Meier P, Finch A, Evan G (2000) Apoptosis in development. Nature 407: 796–801. 11048731 10.1038/35037734

[25] Segura I, Serrano A, De Buitrago GG, Gonzalez MA, Abad JL, et al (2002) Inhibition of programmed cell death impairs in vitro vascular-like structure formation and reduces in vivo angiogenesis. FASEB J 16: 833–841. 12039865 10.1096/fj.01-0819com

[26] Kim KW, Moretti L, Lu B (2008) M867, a novel selective inhibitor of caspase-3 enhances cell death and extends tumor growth delay in irradiated lung cancer models. PLoS One 3: e2275. 18509530 10.1371/journal.pone.0002275 PMC2386548

[27] Moretti L, Kim KW, Jung DK, Willey CD, Lu B (2009) Radiosensitization of solid tumors by Z-VAD, a pan-caspase inhibitor. Mol Cancer Ther 8: 1270–1279. 19417149 10.1158/1535-7163.MCT-08-0893 PMC2888880

[28] Scharner D, Rossig L, Carmona G, Chavakis E, Urbich C, et al (2009) Caspase-8 is involved in neovascularization-promoting progenitor cell functions. Arterioscler Thromb Vasc Biol 29: 571–578. 19122169 10.1161/ATVBAHA.108.182006

[29] Brooks PC, Montgomery AM, Rosenfeld M, Reisfeld RA, Hu T, et al (1994) Integrin alpha v beta 3 antagonists promote tumor regression by inducing apoptosis of angiogenic blood vessels. Cell 79: 1157–1164. 7528107 10.1016/0092-8674(94)90007-8

[30] Schechner JS, Nath AK, Zheng L, Kluger MS, Hughes CC, et al (2000) In vivo formation of complex microvessels lined by human endothelial cells in an immunodeficient mouse. Proc Natl Acad Sci U S A 97: 9191–9196. 10890921 10.1073/pnas.150242297 PMC16844

[31] Hitomi J, Katayama T, Eguchi Y, Kudo T, Taniguchi M, et al (2004) Involvement of caspase-4 in endoplasmic reticulum stress-induced apoptosis and Abeta-induced cell death. J Cell Biol 165: 347–356. 15123740 10.1083/jcb.200310015 PMC2172196

[32] Martinon F, Tschopp J (2004) Inflammatory caspases: linking an intracellular innate immune system to autoinflammatory diseases. Cell 117: 561–574. 15163405 10.1016/j.cell.2004.05.004

[33] Nadiri A, Wolinski MK, Saleh M (2006) The inflammatory caspases: key players in the host response to pathogenic invasion and sepsis. J Immunol 177: 4239–4245. 16982854 10.4049/jimmunol.177.7.4239

[34] Sollberger G, Strittmatter GE, Kistowska M, French LE, Beer HD (2012) Caspase-4 is required for activation of inflammasomes. J Immunol 188: 1992–2000. 22246630 10.4049/jimmunol.1101620

[35] Walsh JG, Cullen SP, Sheridan C, Luthi AU, Gerner C, et al (2008) Executioner caspase-3 and caspase-7 are functionally distinct proteases. Proc Natl Acad Sci U S A 105: 12815–12819. 18723680 10.1073/pnas.0707715105 PMC2529079

[36] Chen M, Wang J (2002) Initiator caspases in apoptosis signaling pathways. Apoptosis 7: 313–319. 12101390 10.1023/a:1016167228059

[37] Kersse K, Vanden Berghe T, Lamkanfi M, Vandenabeele P (2007) A phylogenetic and functional overview of inflammatory caspases and caspase-1-related CARD-only proteins. Biochem Soc Trans 35: 1508–1511. 18031255 10.1042/BST0351508

[38] Ryoo HD, Bergmann A (2012) The role of apoptosis-induced proliferation for regeneration and cancer. Cold Spring Harb Perspect Biol 4: a008797. 22855725 10.1101/cshperspect.a008797 PMC3405855

[39] Means AR, Dedman JR (1980) Calmodulin–an intracellular calcium receptor. Nature 285: 73–77. 6990273 10.1038/285073a0

[40] Rodriguez-Mora OG, LaHair MM, McCubrey JA, Franklin RA (2005) Calcium/calmodulin-dependent kinase I and calcium/calmodulin-dependent kinase kinase participate in the control of cell cycle progression in MCF-7 human breast cancer cells. Cancer Res 65: 5408–5416. 15958590 10.1158/0008-5472.CAN-05-0271

[41] Das SB, Sharma RK (2005) Potential role of calmodulin-dependent phosphodiesterase in human brain tumor (review). Oncol Rep 14: 1059–1063. 16142372

[42] Mukhopadhyay D, Akbarali HI (1996) Depletion of [Ca2+]i inhibits hypoxia-induced vascular permeability factor (vascular endothelial growth factor) gene expression. Biochem Biophys Res Commun 229: 733–738. 8954965 10.1006/bbrc.1996.1873

