# Supplementary figures and images for: Identification of an Effective Early Signaling Signature during Neo-Vasculogenesis In Vivo by Ex Vivo Proteomic Profiling
Source: PLoS One. 2013 Jun 24;8(6):e66909. doi: 10.1371/journal.pone.0066909 (PMC3691264; doi:10.1371/journal.pone.0066909)

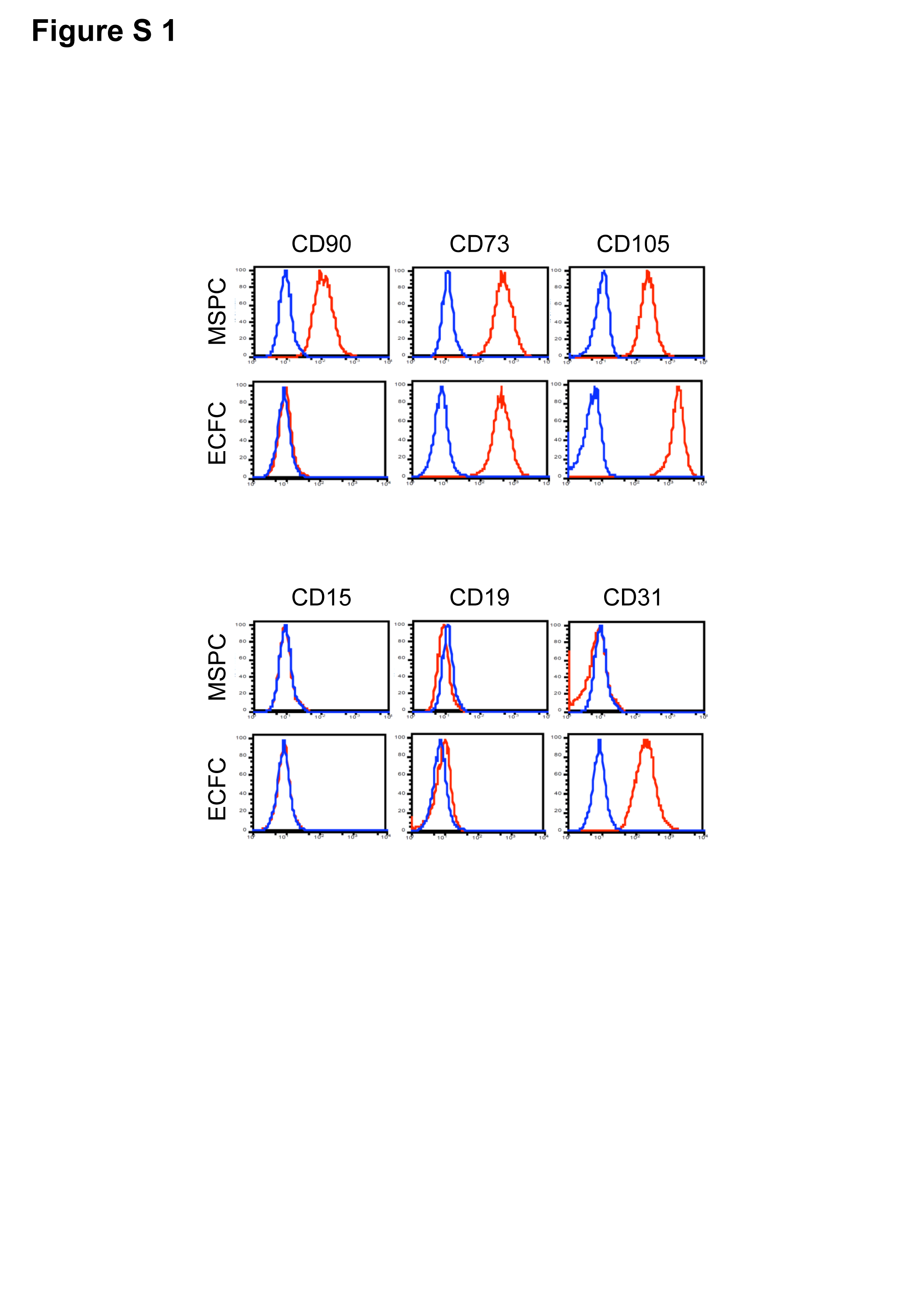

Supplement: Figure S1 — Immune phenotype of culture-expanded cells. Culture-expanded mesenchymal stem/progenitor cells (MSPC; upper rows) and endothelial colony-forming progenitor cells (ECFC; lower rows) as used for co-transplantation were analyzed by flow cytometry for their immune phenotype. Red lines indicate target antibody and blue lines isotype control reactivity. All MSPC used were >95% positive for CD90, CD73 and CD105 fulfilling common ISSCR criteria and lacked reactivity with hematopoietic markers (<2%) as exemplified by CD15 and CD19. ECFC were completely CD90-negative excluding MSPC contamination and their purity was confirmed by CD31 reactivity. One representative example is shown. (TIF) [file pone.0066909.s001.tif]

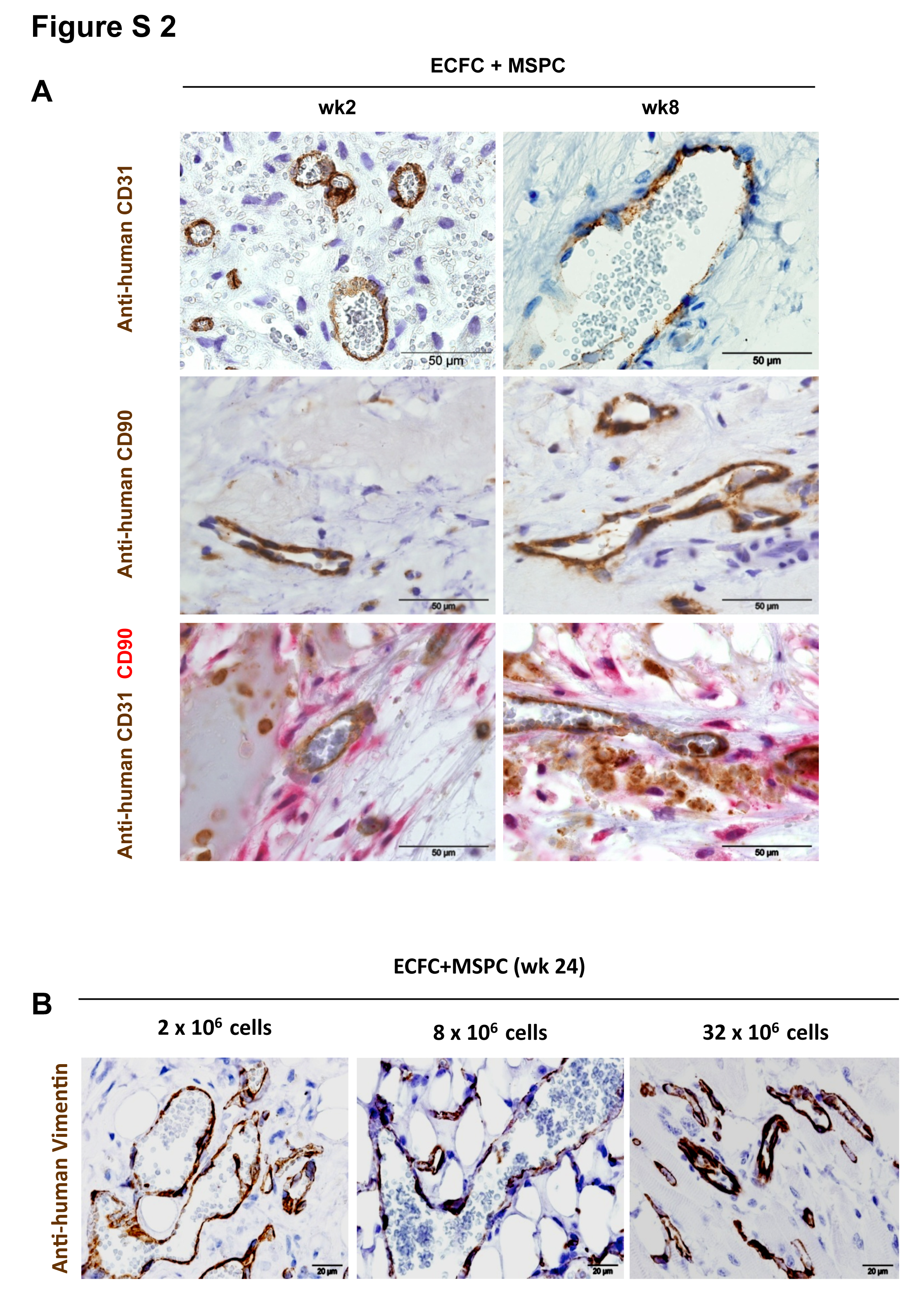

Supplement: Figure S2 — Human vessel formation and persistence. (A) Perfused vessels within ECFC+MSPC co-transplants harvested after two (wk2, left column) and eight weeks (wk8, right column) were visualized by anti-human CD31 (upper row, CD31, brown) and anti-human CD90 immune histochemistry (middle row, brown), verifying contribution of ECFCs to vascular lumen and presence of MSPCs around capillaries. Human CD31/CD90 double staining (lower row, CD31+ in brown, CD90+ in red) depicts intimate localization of MSPCs to ECFCs in vascular structures. Nuclei were counterstained with hematoxylin (blue). (B) Plugs containing umbilical cord derived ECFC+MSPC with a total cell number of 2×10^6^ (left column, n = 2), 8×10^6^ (middle, n = 2) and 32×106 (the latter representing the cell number utilized for obtaining sufficient amounts of protein for antibody array analysis in the initial experiments; right column, n = 2) were harvested 24 weeks after implantation and stained with anti-human vimentin (brown); nuclei are counterstained in blue by hematoxylin. (TIF) [file pone.0066909.s002.tif]

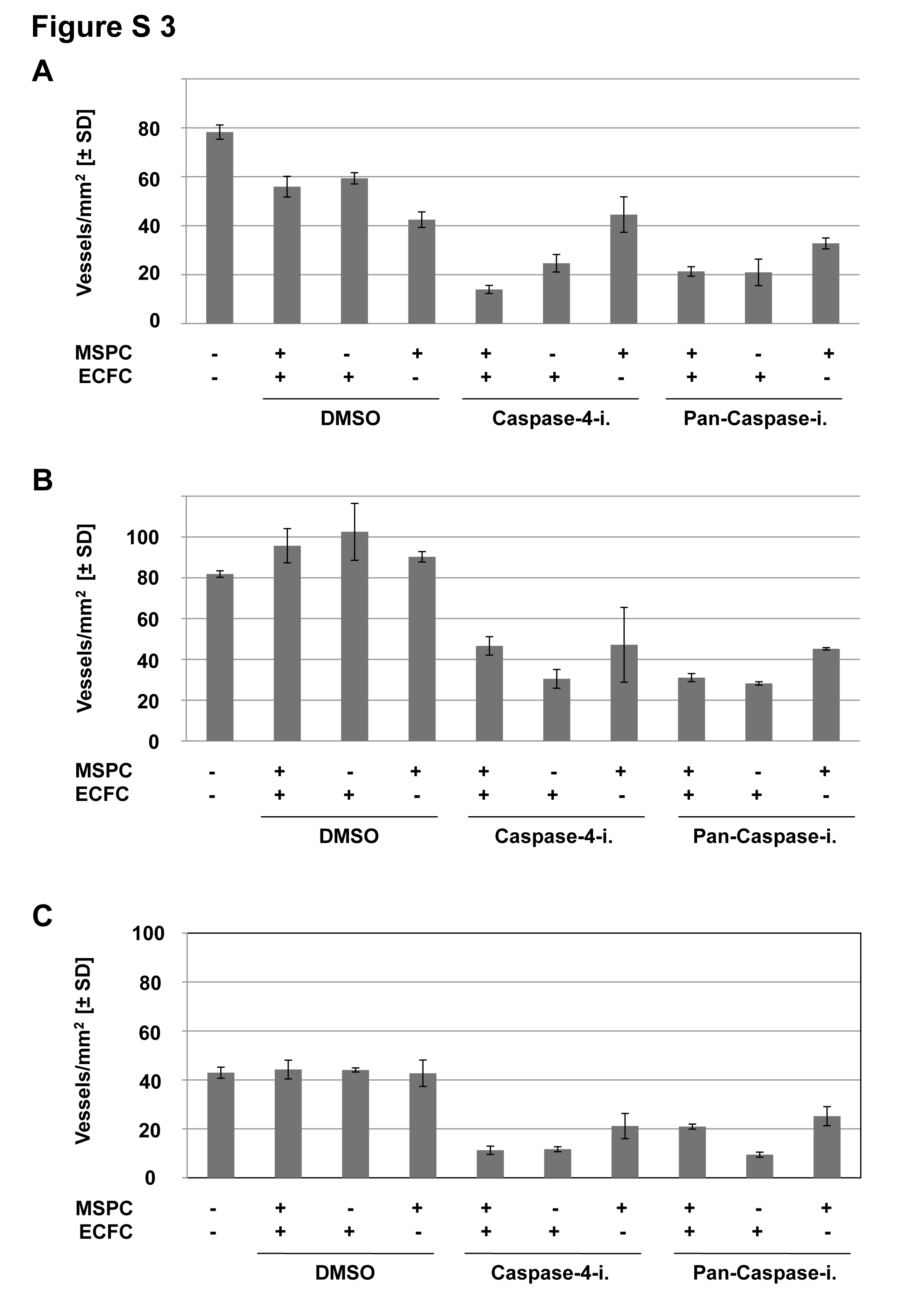

Supplement: Figure S3 — Caspase inhibition hampers human progenitor cell-derived vasculogenesis in vivo. Vascular structure quantification upon DMSO (left panel), caspase-4 (middle panel) and pan-caspase pre-treatment of either MSPC or ECFC or both cell types compared to untreated conditions are shown. (A) Autologous (to each other) MSPC and ECFC were derived from term umbilical cord and culture-expanded before transplantation mimicking the conditions used for the antibody array analysis (n = 2 per condition). (B) White adipose tissue-derived ECFC were co-transplanted with allogeneic umbilical cord blood-derived MSPC to exclude that the inhibitory effect of caspases in this process is restricted to cord-derived cells or specific for autologous progenitor cell pairs (n = 2 per condition). (C) Umbilical cord blood-derived ECFC were co-transplanted with bone marrow-derived MSPC confirming that the inhibitory effect of caspases in this process is not restricted to cord-derived cells and not specific for autologous progenitor cell pairs (n = 2 per condition). (TIF) [file pone.0066909.s003.tif]

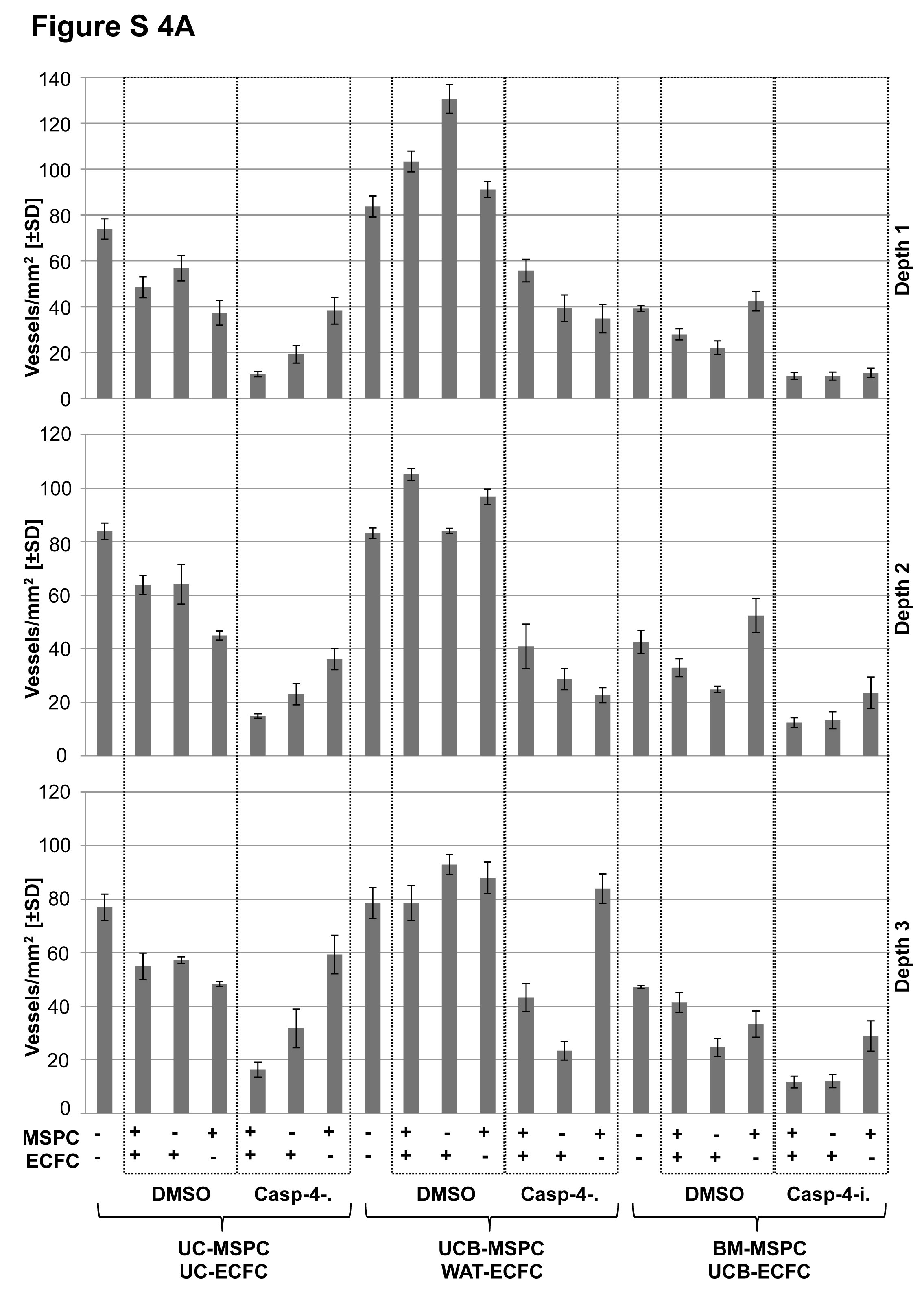

Supplement: Figure S4 — Quantification of vessel density upon caspase inhibition in various depths of the implants. Plugs containing human vessels created by co-transplantation of umbilical cord (UC)-derived ECFC+UC-MSPC (left panels); white adipose tissue (WAT)-derived ECFC+umbilical cord blood (UCB)-derived MSPC (middle panels) and UCB-ECFC+bone marrow (BM)-derived MSPC (right panels). Progenitor cells were either pre-treated (+) or left un-treated (−) with vehicle DMSO or (A) caspase-4 or (B) pan-caspase inhibitor as indicated. Explanted (after 2 weeks) and fixed plugs were cut in three depths with approximately 150 µm intervals and the micro-vessels were counted in different sections. This strategy was used to minimize a hypothetic uneven vessel distribution bias in micro-vessel quantifications within plugs. (TIF) [file pone.0066909.s004.tif]

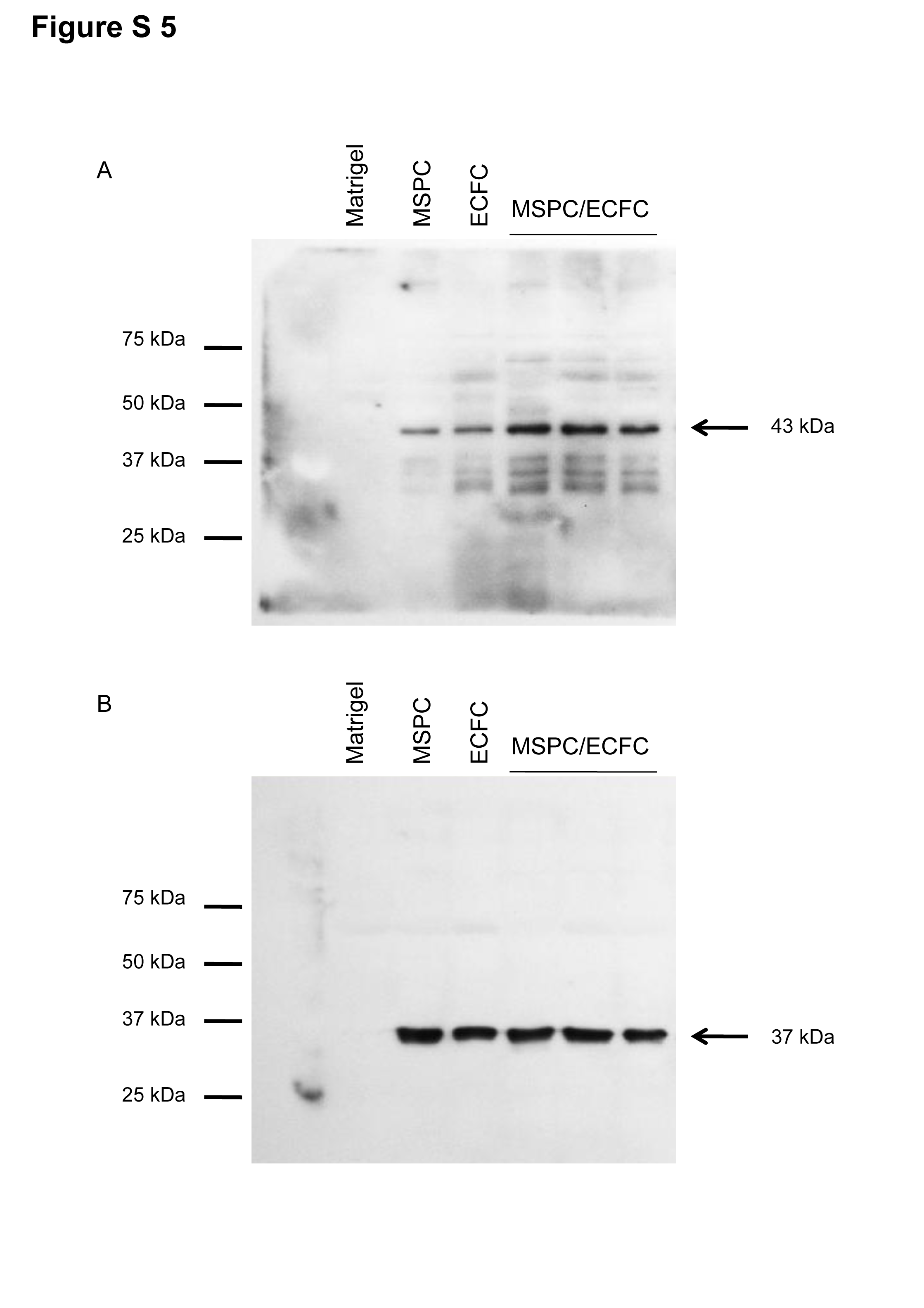

Supplement: Figure S5 — Western blot scan of Caspase-4 and GAPDH analysis. Western blots of Matrix, MSPCs, ECFCs and three independent co-transplants of MSPCs/ECFCs using the protein lysates of the plugs 24 h after transplantation. The Blot was incubated with anti-human caspase-4 (A) or GAPDH antibodies (B). Uncut membrane of the representative results is shown in figure 3. (TIF) [file pone.0066909.s005.tif]

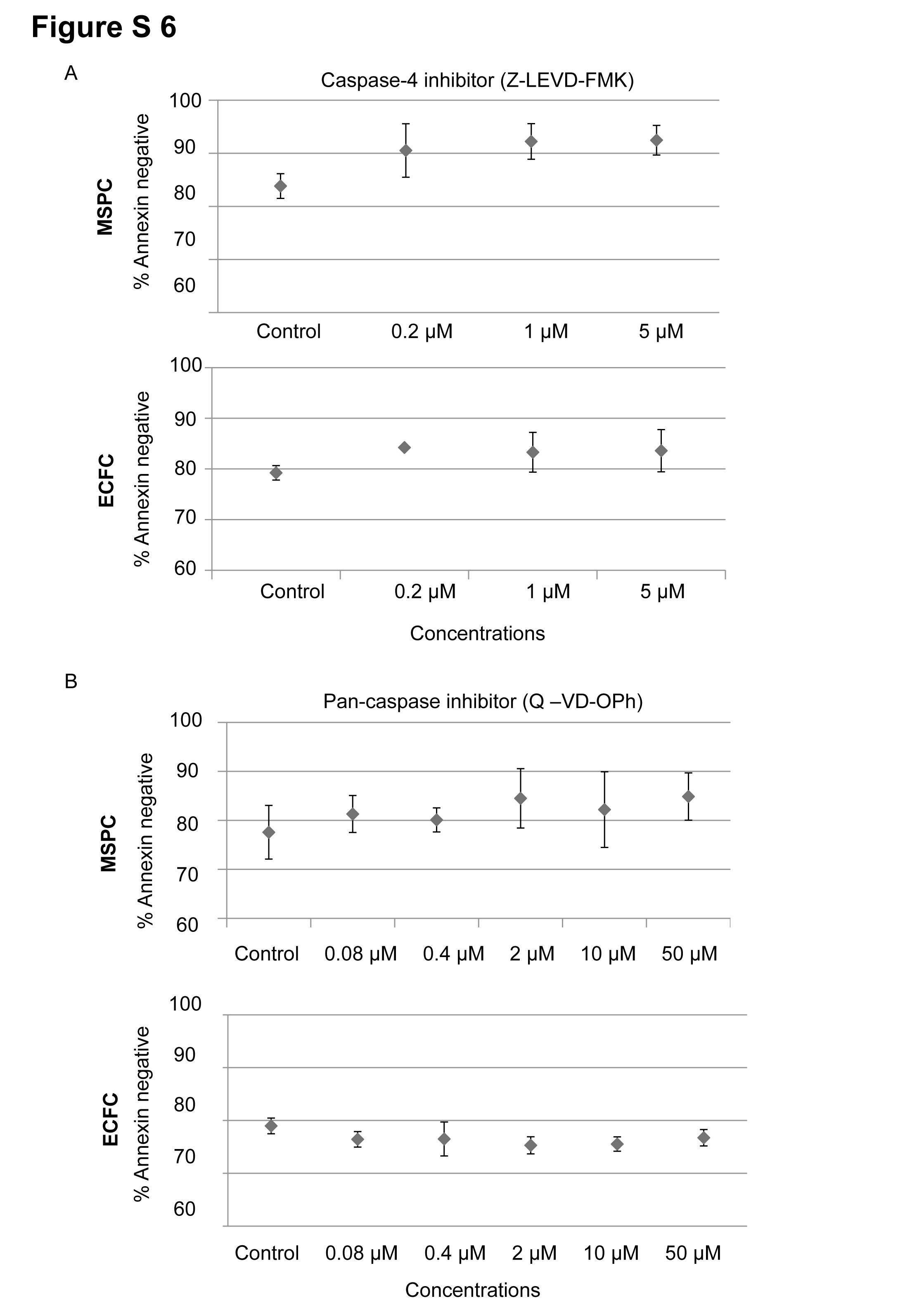

Supplement: Figure S6 — Limited cytotoxicity of caspase inhibitors. MSPC and ECFC were subjected to control (DMSO) or increasing concentration of DMSO-dissolved (A) caspase-4 or (B) pan-caspase inhibitor pre-treatment for 8 h. Cells were washed 2x and Annexin-V-reactivity was determined by flow cytometry to measure apoptotic and dead cells. Percentages of viable annexin negative cells are depicted (mean ± SD of three experiments). (TIF) [file pone.0066909.s006.tif]
