# Supplementary material for: Identification of an Effective Early Signaling Signature during Neo-Vasculogenesis In Vivo by Ex Vivo Proteomic Profiling
Source: PLoS One. 2013 Jun 24;8(6):e66909. doi: 10.1371/journal.pone.0066909 (PMC3691264; doi:10.1371/journal.pone.0066909)
Supplement: Table S1 — Complementary list of signaling molecules expressed in antibody microarrays. (DOCX) [file pone.0066909.s007.docx]

| ***ECFC+MSPC*** | **I. ECFC** | **II. MSPC** | **III. Matrix** | ***IV. in vitro*** |
| --- | --- | --- | --- | --- |
| PKM2 | 🡻 2.06 | 🡻 2.05 |  |  |
| HSP27 | 🡹 1.29 | 🡹 1.89 | 🡹 1.44 |  |
| TTK |  | 🡻 2.12 |  | 🡻 1.25 |
| HO-1 |  |  | 🡹 1.71 | 🡹 1.36 |
| P53 |  |  | 🡹 1.23 |  |
| DAPK1 | 🡹 1.72 |  |  |  |
| DAPK2 | 🡹 1.68 |  |  |  |
| JIK | 🡻 1.43 |  |  |  |
| PKCe | 🡻 1.39 | 🡻 2.67 |  | 🡻 1.45 |
| ILK1 | 🡻 1.33 |  |  |  |
| PP4/A’2 |  | 🡻 2.14 |  |  |
| FAK |  |  |  | 🡹 1.42 |
| FGFR1 |  | 🡹 1.83 |  |  |
| DDR2 |  | 🡻 2.39 |  |  |
| PIk2 | 🡻 1.85 |  | 🡻 1.26 | 🡻 1.34 |
| PIk3 | 🡻 1.63 |  |  | 🡻 1.33 |
| IRAK3 | 🡻 1.66 |  |  |  |
| P38MAPK |  |  |  | 🡹 1.51 |

**Table S1. Complementary list of signaling molecules expressed in antibody microarrays.**

| ***ECFC+MSPC*** | **I. ECFC** | **II. MSPC** | **III. Matrix** | ***IV. in vitro*** |
| --- | --- | --- | --- | --- |
| PSD-95 | 🡻 1.55 |  | 🡻 1.21 | 🡻 1.56 |
| Chk2 |  | 🡹 1.55 |  |  |
| SLK |  | 🡻 2.00 | 🡻 1.26 |  |
| TIK1 |  | 🡻 1.85 |  |  |
| SOD(Cu/Zn) |  | 🡻 1.65 |  |  |
| TAK1 |  | 🡻 1.53 |  |  |
| Rb | 🡻 1.31 |  |  |  |
| PRK 1/2 |  |  | 🡻 1.67 |  |
| CDK10 |  |  | 🡻 1.43 |  |
| HSP105 |  |  | 🡻 1.42 |  |
| MARK |  |  | 🡻 1.39 |  |
| CK1d |  |  | 🡻 1.35 |  |
| Histone H1 |  | 🡹 2.02 |  |  |
| eIF4G | 🡹 1.41 |  |  |  |
| CDK1/2 |  | 🡹 2.27 |  |  |
| PP1/Cg |  |  | 🡻 1.28 |  |
| Vrk1 |  |  |  | 🡻 1.42 |
| Nrf2 |  |  |  | 🡹 1.25 |
|  |  |  |  |  |

**Footnote Table S1.**

See Footnote Table 1 for the table description.

**Table abbreviations**

**PKM2:** Pyruvate kinase, isozymes M1/M2; **Hsp27:** Heat shock 27 kDa protein beta 1 (HspB1); **TTK:** Dual specificity protein kinase; **HO-1:** Hemeoxygenase1; **P53:** Tumor suppressor protein p53 (antigen NY-CO-13); **DAPK1:** Death-associated protein kinase 1; **DAPK2:** Death-associated protein kinase 2; **JIK (TAO3):** STE20-like protein-serine kinase; **PKCe:** Protein-serine kinase C epsilon; **ILK1:** Integrin-linked protein-serine kinase 1; **PP4/A’2:** Protein-serine phosphatase 4 - regulatory subunit (PPX/A'2); **FAK:** Focal adhesion protein-tyrosine kinase; **FGFR1:** Fibroblast growth factor receptor-tyrosine kinase 1; **DDR2 (Tyro10):** Neurotrophic receptor-tyrosine kinase of discoidin domain receptor family, member 2 precursor; **PIK2:** Polo-like protein kinase 2 (serum -inducible kinase (SNK)); **PIK3:** Polo-like protein kinase 3 (cytokine- inducible kinase (CNK)); **IRAK3:** Interleukin 1 receptor-associated kinase 3; **p38MAPK:** Mitogen-activated protein-serine kinase p38; **PSD-95:** Disks large homolog 4; **Chk2:** Checkpoint protein-serine kinase 2; **SLK:** STE20-like protein-serine kinase; **Tlk1:** Tousled-like protein-serine kinase 1; **SOD (Cu/Zn):** Superoxide dismutase 1; **TAK1:** TGF-beta-activated protein-serine kinase 1; **Rb:** Retinoblastoma-associated protein 1; **PRK1/2:** Protein kinase C-related protein-serine kinase 1/2; **CDK10:** Cyclin-dependent protein-serine kinase 10; **Hsp105:** Heat shock 105 kDa protein; **MARK:** MAP/microtubule affinity-regulating protein-serine kinase 1; **CK1d:** Casein protein-serine kinase 1 delta; **Histone H1:** Histone H1 phosphorylated; **eIF4G:** Eukaryotic translation initiation factor 4 gamma 1; **CDK1/2:** Cyclin-dependent protein-serine kinase 1/2; **PP1/Cg:** Protein-serine phosphatase 1-catalytic subunit-gamma isoform; **Vrk1:**Vaccinia related protein-serine kinase 1; **Nrf2:** Nuclear factor erythroid 2-related factor 2.
